# Genetic Diversity and Symbiotic Phenotype of Hairy Vetch Rhizobia in Japan

**DOI:** 10.1264/jsme2.ME15184

**Published:** 2016-05-03

**Authors:** Kun Yuan, Hiroki Miwa, Maki Iizuka, Tadashi Yokoyama, Yoshiharu Fujii, Shin Okazaki

**Affiliations:** 1 Graduate School of Agriculture, Tokyo University of Agriculture and TechnologySaiwaicho 3–5–8, Fuchu, Tokyo 183–8509Japan

**Keywords:** hairy vetch (*Vicia villosa*), Rhizobia, nodulation

## Abstract

Hairy vetch (*Vicia villosa* Roth) is a leguminous crop widely used as green manure and a cover crop in Japan. It exhibits strong weed-suppressing activity, high resistance to insect pests, and the ability to fix nitrogen through symbiotic interactions with soil bacteria known as rhizobia. Few studies have investigated the rhizobia that form nodules on hairy vetch in Japan, and the biological resources available for selecting high nitrogen-fixing rhizobia are limited. In the present study, we isolated 110 hairy vetch rhizobia from 13 different areas in Japan. Based on their 16S rRNA gene sequences, 73% of the isolates were identified as *Rhizobium leguminosarum*. A comparative analysis of *nodC* and 16S rRNA gene phylogenies revealed that several isolates possessed congruent *nodC* sequences despite having divergent 16S rRNA gene sequences, suggesting that the horizontal transfer of *nod* genes occurred during the evolution of rhizobia. Inoculation tests showed that isolates closely related to *R. leguminosarum* had better plant growth-promoting effects than other strains, thereby providing a promising agricultural resource for inoculating crops.

Nitrogen fixation by root nodule rhizobia plays an important role in supplying nitrogenous nutrients to leguminous plants. Rhizobia form organs called nodules on host plant roots in which atmospheric diatomic nitrogen is fixed into ammonia, and this is then utilized directly for plant growth. Large amounts of chemical fertilizers have recently been used in agriculture to meet increasing demands for crop production; however, this occasionally causes serious environmental issues. Therefore, the wider application of leguminous crops together with symbiotic rhizobia to agricultural practice is one of the more promising ways by which to increase production in a sustainable manner.

Symbiosis between legumes and rhizobia is highly specific (16). A group of rhizobia may only establish symbiosis with a certain group of legumes. This host specificity may occur at the level of nodule formation and the efficiency of nitrogen fixation (24). Host specificity is determined by legume and rhizobial genotypes. In order to improve the agronomic potential of root nodule symbiosis, it is important to understand the genetic diversity of rhizobia and apply appropriate rhizobia to a particular leguminous crop. This host specificity significantly affects the biodiversity of rhizobia in the natural environment because the symbiotic relationship also has great benefits for rhizobia by providing a physical site to grow and a carbon supply from the host plant. In addition, the geographical region defines the diversity of rhizobia because factors such as the climate and soil properties contribute to the soil environment in which rhizobia live.

Hairy vetch (*Vicia villosa*) is a leguminous plant that is widely used as green manure or a cover crop in Japan (6). It has the ability to provide up to 20–25 kg N 10 a^−1^ to the soil as a result of symbiosis with rhizobia. It survives at temperatures as low as −20°C, tolerates a wide range of soil pH values (6, 8), and exerts strong weed-suppressing effects by accumulating the allelochemical, cyanamide (6, 10). Hairy vetch is nodulated specifically by *Rhizobium leguminosarum* symbiovar *viciae*, which is the same rhizobial species that nodulates peas (*Pisum sativum*), faba beans (*V. faba*), and common vetch (*V. sativa*) (13). Previous studies demonstrated that an inoculation with *R. leguminosarum* symbiovar *viciae* improved hairy vetch production (2). However, few studies have investigated rhizobia that nodulate hairy vetch in Japan, and the biological resources available for selecting high nitrogen-fixing rhizobia are limited.

The aim of the present study was to assess the genetic diversity of hairy vetch rhizobia in Japan and evaluate symbiotic phenotypes. We isolated rhizobial strains from various locations in Japan with different climates. This study provides new insights into the ecological aspects of rhizobial biodiversity, as well as providing novel rhizobial isolates as genetic resources with diverse characteristics adapted to specific environments.

## Materials and Methods

### Sampling sites

**Fig. 1:**
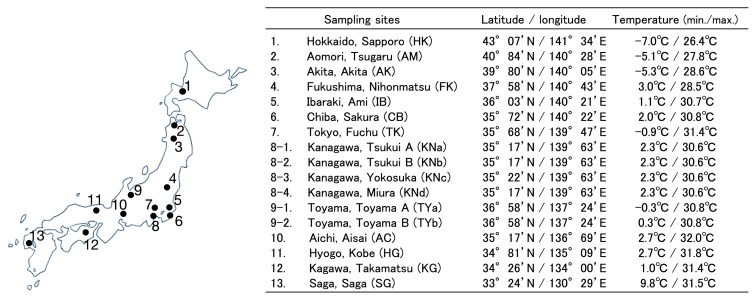
Sampling sites of hairy vetch. Hairy vetch plants were collected from the 13 sites marked with black dots.

Hairy vetch plants were sampled from 13 sites throughout Japan: Hokkaido, Aomori, Akita, and Fukushima in northern Japan, Ibaraki, Chiba, Tokyo, Kanagawa, Toyama, and Aichi in central Japan and Hyogo, Kagawa, and Saga in southern Japan (Fig. 1). Hokkaido was the northernmost site and has a humid, subarctic climate with a relatively low level of rainfall. Saga was the most south-western site and has a warm climate. Hairy vetch plants were collected between May and July 2013 and 2014. Five to ten plants were sampled from each sampling site.

### Isolation of rhizobia from root nodules of hairy vetch

Nodules were detached from hairy vetch roots and surface sterilized using 70% ethanol for 30 s and 1% sodium hypochlorite for 30 s, then rinsed three times with sterile water. The nodules were then crushed in 0.5 mL 0.9% sodium chloride, the suspension was streaked on Rhizobium-defined medium (RDM) agar plates (20), and the plates were incubated at 28°C for 3–7 d. Single colonies were picked up and used for further analyses. Only one strain was isolated from each plant; therefore, 5 to 10 strains were isolated from each sampling site.

### DNA amplification and sequencing and phylogenetic analyses

Genomic DNA was extracted from isolates using Wizard Genomic DNA purification kits (Promega, WI, USA). The 16S ribosomal RNA (rRNA) genes were amplified using a polymerase chain reaction (PCR) with 10 ng purified DNA, LA taq (TaKaRa, Tokyo, Japan), and the primers 16AF (5′-AACTGAAGAGTTTG ATCMTGGCTCAG-3′) and 1492R (5′-TACGGYTACCTTGTTA CGACTT-3′) under the following conditions: preheating at 95°C for 1 min; 30 cycles of 98°C for 10 s and 68°C for 90 s; and a final extension at 72°C for 10 min. The *nodC* genes were amplified similarly using the primers nodCF (5′-AYGTHGTYGAYGACG GTTC-3′) and nodCI (5prime;-CGYGACAGCCANTCKCTATTG-3prime;) under the following conditions: preheating at 95°C for 2 min; 30 cycles of 95°C for 30 s, 55°C for 30 s, and 72°C for 1 min; and a final extension at 72°C for 5 min. The *nifH* genes were amplified similarly using the primers nifHF (5prime;-TACGGNAARGGSGGNAT CGGCAA-3prime;) and nifHI (5prime;-AGCATGTCYTCSAGYTCNTCCA-3prime;) under the following conditions: preheating at 95°C for 2 min; 30 cycles of 95°C for 30 s, 57°C for 30 s, and 72°C for 1 min; and a final extension at 72°C for 5 min. The *recA* genes were amplified similarly using the primers recAF (5prime;-CGKCTSGTAGAGGAYA AATCGGTGGA-3prime;) and recAR (5prime;-CGRATCTGGTTGATGAAG ATCACCAT-3prime;) under the following conditions: preheating at 95°C for 5 min; 30 cycles of 94°C for 45 s and 50°C for 60 s; and a final extension at 74°C for 90 s. The *atpD* genes were amplified similarly using the primers atpDF (5prime;-SCTGGGSCGYATCMTGAACGT-3prime;) and atpDR (5prime;-GCCGACACTTCCGAACCNGCCTG-3prime;) under the following conditions: preheating at 95°C for 3 min; 14 cycles of 95°C for 30 s, 65°C (decrease 0.5°C per cycle) for 30 s, and 72°C for 1 min; 21 cycles of 95°C for 30 s, 58°C for 30 s, 72°C for 1 min; and a final extension at 72°C for 3 min. PCR products were purified with FastGene Gel/PCR Extraction Kits (Nippon Genetics, Tokyo, Japan.) and directly sequenced using primers for PCR. The presence of genetic variations between copies of the same gene was analyzed by the peak intensities in raw sequence data. In order to distinguish between PCR errors and genetic variations, genetic variations were only counted when there were two or more peaks of significant intensities at the same position in the reads from both directions. However, we did not observe multiple peaks of significant intensities in the present study. Partial nucleotide sequences from 16S rRNA, *nodC*, *nifH*, *recA*, and *atpD* were used to estimate the phylogenetic relationships of the isolates. The sequences obtained were BLAST-searched in the GenBank database in order to identify the most closely related sequences. All of the sequences obtained and the reference sequences were aligned using the ClustalW program. Phylogenetic trees were constructed using MEGA software ver.6 (23). A consensus tree was generated using the neighbor-joining method, the Kimura 2-parameter model, and 1,000 bootstrap replications.

### Nodulation test

Rhizobial strains were tested for their nodulation ability and symbiotic performance on hairy vetch. Hairy vetch seeds (*V. villosa*, cultivar Fujiemon) were obtained from Snow Brand Seed (Tokyo, Japan). Seeds were surface-sterilized using 3% NaClO (Wako Pure Chemical Industries, Osaka, Japan) for 2 min and washed 5 times with sterile water to remove the disinfectant. Seeds were transferred aseptically to 1% water agar plates and allowed to germinate at 25°C for 3 d in the dark. Seedlings were then transferred to a plant box (CUL-JAR300; Iwaki, Tokyo, Japan) containing sterile vermiculite (Hirukon S, Hiruishi Kagaku Kogyo, Osaka, Japan) watered with B&D nitrogen-free medium (1). Plants were then inoculated with rhizobial strains at a concentration of 10^7^ cells (2 mL plant^−1^). Plants were grown in a plant growth chamber (LPH-410SP; Nihonika Osaka, Japan) at 25°C on a 16-h light/8-h dark cycle. Nodule weight and fresh plant weight were measured 3 weeks after inoculation. Each bacterial isolate was tested in triplicate in the nodulation tests. Nodule structures were examined using an Olympus SZX Research Stereomicroscope System (Olympus, Tokyo, Japan).

### Nucleotide sequence accession numbers

DNA sequences were deposited in the DNA Data Bank of Japan (DDBJ) under accession numbers LC042344 to LC042453 (16S rRNA), LC042236 to LC042343 (*nodC*), LC125270 to LC125289 (*nifH*), LC076297 to LC076321 (*atpD*), and LC076322 to LC076346 (*recA*).

## Results and Discussion

### Isolation and phylogenetic analysis of hairy vetch rhizobia

**Fig. 2:**
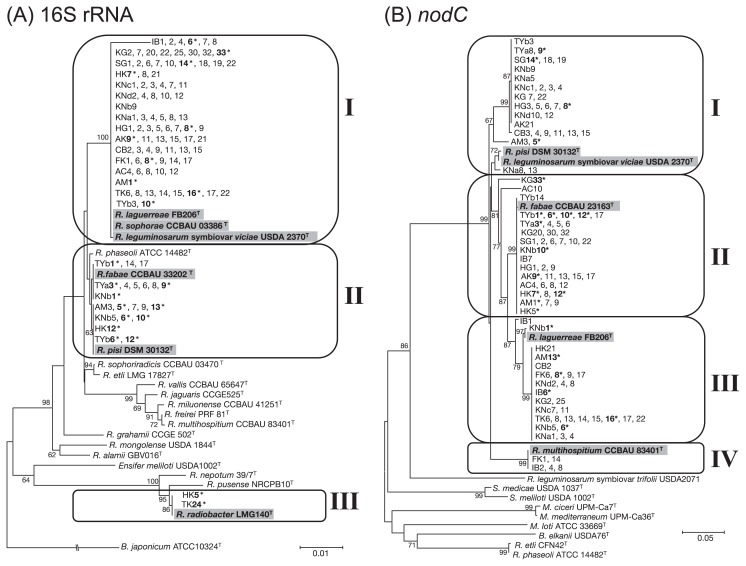
Phylogenetic trees for 16S rRNA (A) and *nodC* (B) gene sequences of isolates and related strains. (A) Phylogenetic trees of the 16S rRNA gene were constructed using a 1370-bp partial nucleotide sequence from 110 isolates and the type strains of each species belonging to different genera. (B) Phylogenetic trees of the *nodC* gene were constructed using an 835-bp partial nucleotide sequence from 109 isolates and the type strains of each species belonging to different genera. Bootstrap values are shown as percentages from 1,000 replicates. Strains used for nodulation tests are marked with asterisks.

Hairy vetch plants were collected from 13 sites in Japan (Fig. 1) and 110 strains were isolated from nodules. A phylogenetic analysis using the 16S rRNA gene showed that the isolates clustered into three groups that were closely related to *R. leguminosarum* (group I, 87 isolates), *R. fabae*/*R. pisi* (group II, 21 isolates), or *R. radiobacter* (group III, 2 isolates) (Fig. 2A). The largest cluster (group I) included isolates from all 13 sampling sites, suggesting that strains related to *R. leguminosarum* were the most common symbionts of hairy vetch and were widely distributed in Japan. This was consistent with previous findings showing that the *Vicia* genus establishes symbiotic relationships with *R. leguminosarum* in different areas worldwide, including Europe, Asia, America, and Africa (3, 11, 14). Strains closely related to *R. pisi* were found in a distinct geographical area in northern Japan while those closely related to *R. fabae* were predominantly found in Toyama prefecture. *R. pisi* and *R. fabae* have both been reported to nodulate *Vicia* spp., although with different host specificities. *R. pisi* was initially isolated from *P. sativum* and was also reported to be able to nodulate *Phaseolus vulgaris* and *Trifolium repens* (18). *R. fabae* was isolated from *V. faba* (11). The isolates closely related to *R. pisi* and *R. fabae* may only occasionally nodulate hairy vetch and have better host legumes in their habitat. Although some strains belonging to *Ensifer*, *Bradyrhizobium*, and *Mesorhizobium* have been reported to establish symbiosis with *Vicia* species (12), we did not detect any of these rhizobia in this study, suggesting that these strains are less adaptive and competitive in Japanese environments.

Regarding further species identification, two housekeeping genes, *recA* and *atpD* were sequenced for 25 representative isolates that were selected based on sampling sites and taxonomic positions determined by a 16S rRNA phylogenetic analysis. The results obtained supported the overall phylogenic positions of these isolates based on 16S rRNA, showing that the isolates in group I in the 16S rRNA phylogeny were clustered in group I in the *atpD* phylogeny and in group II in the *recA* phylogeny. Similarly, the isolates in group II in 16S rRNA phylogeny were clustered in group II in the *atpD* phylogeny and in group I in the *recA* phylogeny (Fig. S1A and B).

The *nodC* gene is essential for nodulation in most rhizobia and has been used as a genetic marker for investigating symbiotic diversity and host specificity in rhizobia (19, 22). The *nodC* genes from 109 isolates were sequenced and used for a phylogenetic analysis. *R. radiobacter* strain TK24 was excluded from the analysis because the *nodC* gene was not amplified by our PCR. The isolates clustered into four groups that were closely related to *R. fabae* (group II, 42 isolates), *R. laguerreae* (group III, 30 isolates), *R. leguminosarum* symbiovar *viciae*/*R. pisi* (group I, 32 isolates), or *R. multihospitium* (group IV, 5 isolates) (Fig. 2B). Previous studies have reported that hairy vetch is mainly nodulated by *R. leguminosarum* symbiovar *viciae*. However, *R. fabae*, *R. laguerreae*, and *R. multihospitium* have also been reported to nodulate *Vicia* spp. including hairy vetch (9, 11, 21). Our results confirmed this previous classification of host specificity. In addition, *nodC* genes in the hairy vetch isolates from Japan formed a large cluster, which was clearly separated from that of *R. leguminosarum* symbiovar *viciae*, suggesting that the Japanese isolates had a common geographical origin.

In further investigations, we sequenced the *nifH* gene of 25 representative isolates that were used for *atpD* and *recA* analyses. We failed to sequence 5 isolates (AK9, CB2, KG33, HK5, and TK24) and have not yet identified the reason for this. The *nifH* gene encodes a dinitrogenase reductase subunit (5) and is the most widely used biomarker to study the ecology and evolution of nitrogen-fixing bacteria. The results obtained showed that these isolates clustered into 3 groups in the phylogenetic tree, the overall structure of which was similar to the *nodC* phylogeny (Fig. S2).

The phylogenies derived from the 16S rRNA and *nodC* (and *nifH*) genes exhibited different structures. For example, strains TYb3, TYa8, and AM3 possessed congruent *nodC* genes (Fig. 2B), whereas these strains were in different groups based on their 16S rRNA gene sequences (groups I, II, and III in Fig. 2A). This result suggested that these isolates acquired *nodC* genes by lateral gene transfer. Symbiotic capacity may have evolved by the exchange of symbiotic genes and genomic rearrangements within rhizobial strains, accompanying the adaptation and domestication of hairy vetch.

Geographical distance has been shown to affect the diversity of rhizobia (4). In order to assess the geographical distribution and diversity of *Vicia*-nodulating rhizobia, we performed a biogeographical analysis using the *nodC* sequences of Japanese hairy vetch isolates and those deposited in the database as *Vicia*-nodulating isolates. In the phylogenetic tree, most of the clades contained both Japanese/Asian isolates and European isolates (Fig. S3). The clade of *R. laguerreae* contained isolates from diverse areas of the world. These results suggest that geographical factors did not contribute to the separation of bacterial symbionts in *Vicia* species or that extensive lateral gene transfer occurred and superimposed genetic diversity caused by geographical separation. Despite the overall co-existence of Japanese and European isolates, the most closely related strains of Japanese isolates were often those from China and South Korea, indicating that the origin of *Vicia*-nodulating rhizobia in Japan was East Asia.

In the present study, we explored the genetic diversity of hairy vetch rhizobia collected from various locations with different climates. However, soil chemical properties also greatly influence the diversity of rhizobia. Further studies using a soil analysis of hairy vetch sampling sites are needed in order to elucidate the relationship between the soil chemical properties and genetic diversity of hairy vetch rhizobia.

### Symbiotic properties

**Fig. 3:**
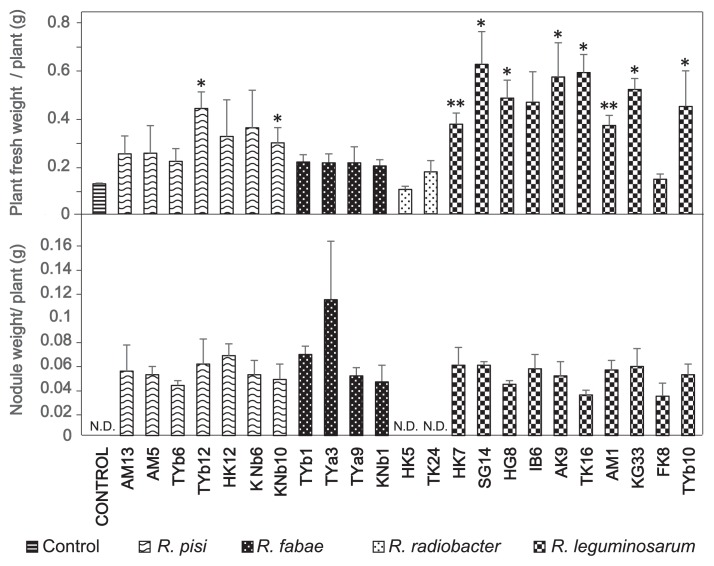
Growth and nodulation of hairy vetch plants inoculated with different rhizobial isolates. The fresh weight of whole plants was measured 3 weeks after the inoculation. Nodulation tests were performed at least three times. Values shown are the means of at least 3 plants and error bars represent SDs. Results for each strain were compared to uninoculated controls using the Student’s *t*-test; *P<0.05, **P<0.01. Nodule weights from hairy vetch plants were measured 3 weeks after the inoculation. Nodulation tests were performed at least three times. Values shown are means of at least 3 plants and error bars represent SDs. Results for each strain were compared to uninoculated controls using the Student’s *t*-test; *P<0.05, **P<0.01. N.D., not detected.

The symbiotic properties of the isolates were tested using 23 representative strains selected on the basis of the 16S rRNA phylogeny (Fig. 2A). With the exception of two isolates (HK5 and TK24), most isolates formed nodules and promoted plant growth (Fig. 3). Among these isolates, those most closely related to *R. leguminosarum* strains were the most effective at promoting plant growth. SG14, AK9, TK16, and KG33 exerted 5-fold stronger effects on hairy vetch fresh weight than the control. The two isolates (HK5 and TK24), which were closely related to *R. radiobacter*, did not form any nodules on hairy vetch. Although we did not determine whether these isolates were infected, *R. radiobacter* strains have been reported as endophytes (25), suggesting that HK5 and TK24 associate with hairy vetch as endophytes.

**Fig. 4:**
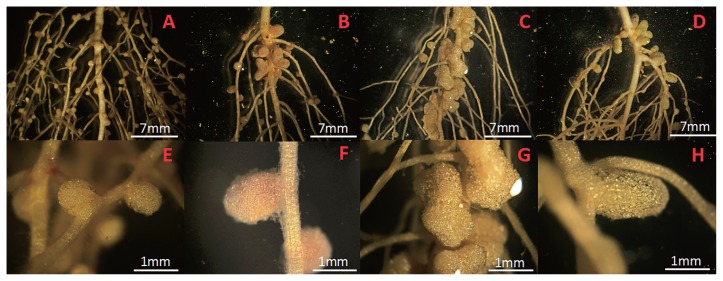
Photographs of nodules on hairy vetch roots 3 weeks after an inoculation with different rhizobial isolates. (A, E) *R. pisi* isolate AM5; (B, F) *R. leguminosarum* isolate TYb10; (C, G) *R. fabae* isolate TYa3; (D, H) *R. leguminosarum* isolate FK8.

Strain TYa3, related to *R. fabae*, formed the largest and heaviest nodules of all the isolates tested (Fig. 3, Fig. 4C and G), whereas *R. leguminosarum* strains induced a few large nodules (Fig. 4B and F) and *R. pisi* strains formed many small nodules (Fig. 4A and E). However, the total weight of the nodules per plant was not significantly different between the different isolates (Fig. 3). These results may have been due to the plant regulating nodule formation in order to balance nitrogen demand at the cost of forming and maintaining nodules.

The structure of the nodules that form on hairy vetch plants was examined in order to investigate the infectious properties of the isolates. As shown in Fig. 4, four strains induced atypical nodules that were markedly different from the nodules formed by other isolates. AM5 (Fig. 4A and E) belonged to the *R. pisi* group and formed many small round nodules that were white, indicating that the nodules exhibited lower nitrogen-fixing activity. The *R. fabae* group isolates formed similar nodules to those formed by *R. pisi*. TYb10 (Fig. 4B and F) in the *R. leguminosarum* group formed a few large heart-shaped nodules that were light pink. TYa3 (Fig. 4C and G) in the *R. fabae* group formed popcorn-like nodules that were also white. FK8 (Fig. 4D and H) in the *R. leguminosarum* group formed rod-shaped, indeterminate types of nodules. These nodules were greenish in color, suggesting early senescence and poor efficiency in these nodules. The number, size, and structure of the nodules formed by the isolates varied; however, they were all originally isolated from large mature nodules on hairy vetch plants. This may reflect diversity in their symbiotic genes. Several rhizobial factors have been reported to affect nodulation efficiency. Among these are the nodulation genes involved in the synthesis of and modifications to the Nod factors that induce nodule organogenesis in host legumes (4). In addition, differences in nodule structures in some isolates may be the result of differences in the production and secretion of phytohormones, such as auxin and cytokinin, by rhizobia (15, 17) or from differences in the infection patterns of rhizobia for host plants (7).

In symbiotic phenotype tests, no significant difference was observed in nodule weight, whereas a marked difference was noted in plant weight. This result strongly suggests a difference in the nitrogen-fixing ability of different hairy vetch rhizobia. The *nif* and *fix* genes, which encode components of nitrogenase and its regulatory proteins (5), may not function equally efficiently in all nodules. The most efficient nitrogen-fixing rhizobial strains belonged to the *R. leguminosarum* group in the 16S rRNA classification, but were in different species groups when classified according to their *nodC* or *nifH* genes. Therefore, a relationship did not appear to exist between symbiotic performance and symbiosis-related genes, but it may be related to 16S rRNA and house-keeping genes.

## Conclusions

We herein describe for the first time the genetic diversity of hairy vetch rhizobia in Japan. We found that *R. leguminosarum* is widely distributed all over Japan and exerts high growth-promoting effects on hairy vetch. Phylogenetic analyses using 16S rRNA, *atpD*, *recA*, *nodC*, and *nifH* genes suggest the horizontal gene transfer of symbiosis-related genes to rhizobia from different backgrounds. This study also provides biological resources for selecting effective rhizobia for agriculture.

## Supplementary Material
