# Supplementary material for: Genetic Diversity and Symbiotic Phenotype of Hairy Vetch Rhizobia in Japan
Source: Microbes Environ. 2016 May 3;31(2):121–6. doi: 10.1264/jsme2.ME15184 (PMC4912146; doi:10.1264/jsme2.ME15184)
Supplement: Supplementary file 1 [file 31_121_s1.pdf]

Fig. S1. Phylogenetic trees for *atpD* (A) and *recA* (B) gene sequences of isolates and related strains. (A) The phylogenetic tree of the *atpD* gene was constructed using a 350-bp partial nucleotide sequence from 25 representative isolates and the type strains of each species belonging to different genera. (B) The phylogenetic tree of the *recA* gene was constructed using a 565-bp partial nucleotide sequence from 25 isolates and the type strains of each species belonging to different genera. Bootstrap values are shown as percentages from 1,000 replicates. Strains used for nodulation tests are marked with asterisks.

Fig. S2. Phylogenetic tree for *nifH* gene sequences of *Vicia*-nodulating isolates and related strains. The phylogenetic tree was constructed using a 689-bp partial nucleotide sequence from 20 isolates and the type strains of each species belonging to different genera. Strains used for nodulation tests are marked with asterisks.

Fig. S3. Phylogenetic tree for *nodC* gene sequences of *Vicia*-nodulating isolates and related strains. The phylogenetic tree was constructed using an 835-bp partial nucleotide sequence. The GenBank accession numbers of the *nodC* gene sequence and isolation sites are shown. Colors correspond to isolation sites: Asia (red), Europe (green), Africa (blue), Oceania (brown), and South America

(purple). Type strains are shown in black. Bootstrap values are shown as percentages from 1,000 replicates. Strains used for nodulation tests are marked with asterisks.

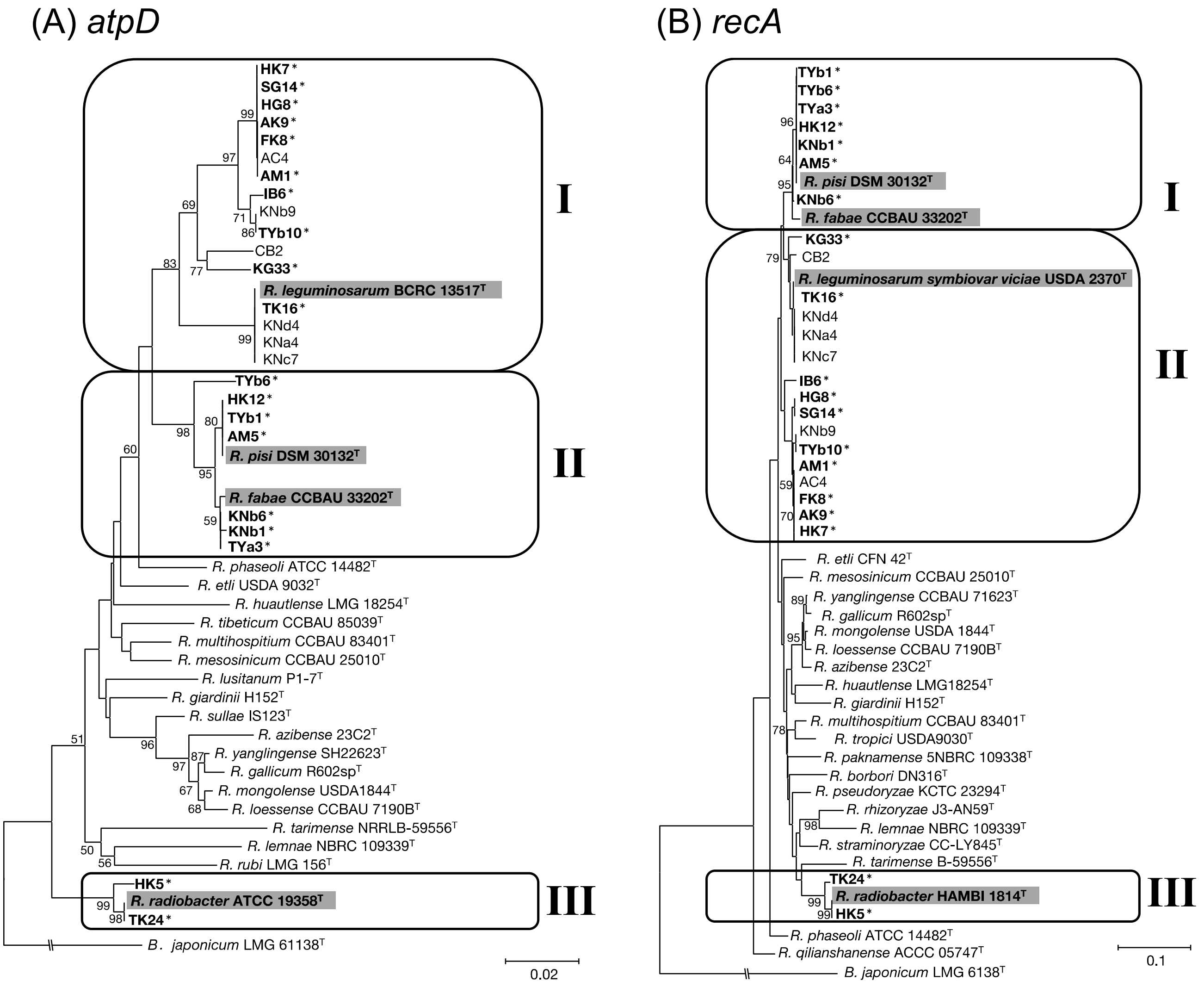

Fig. S1.
(Kun Yuan)

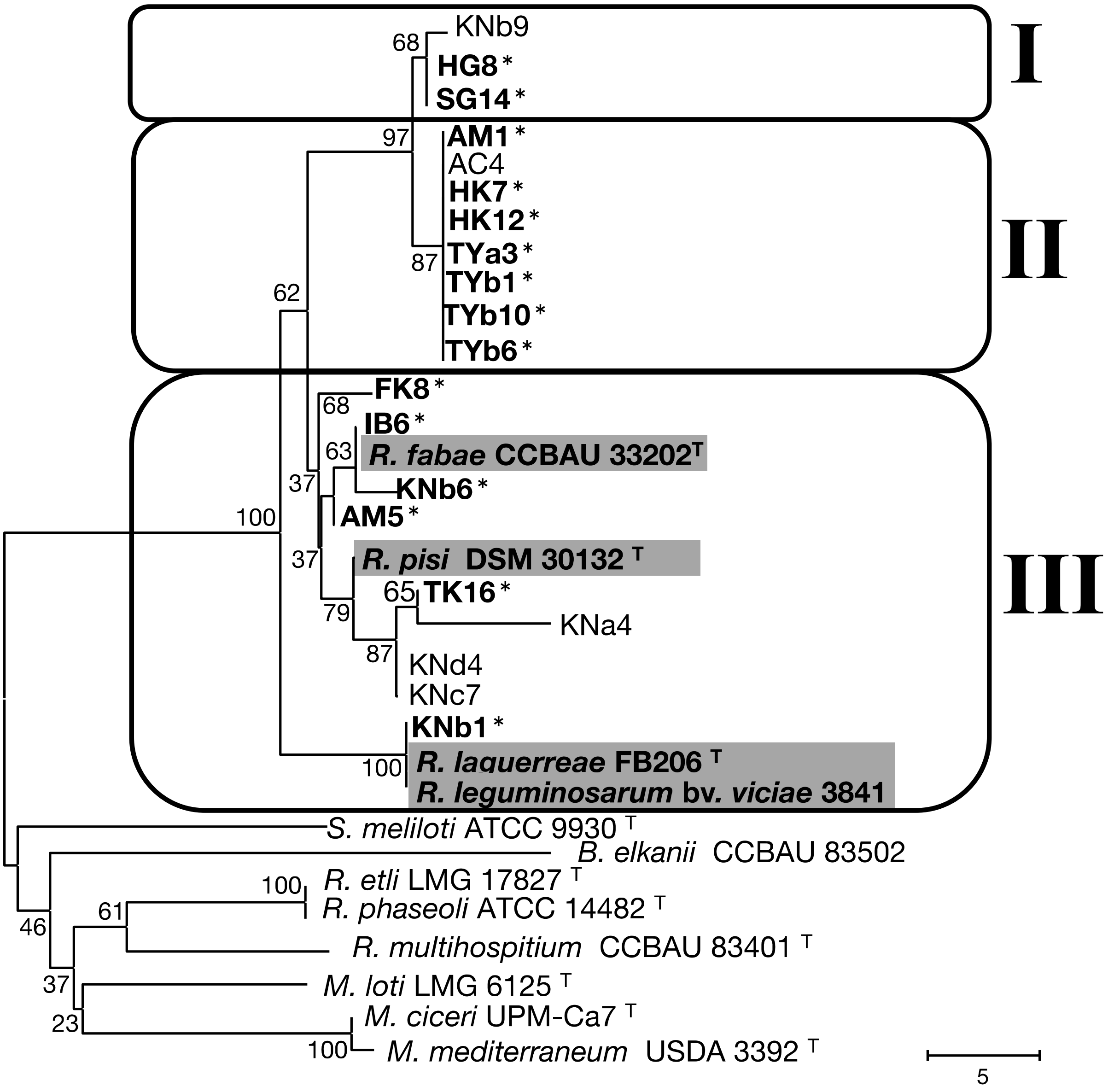

Fig. S2.

(Kun Yuan)

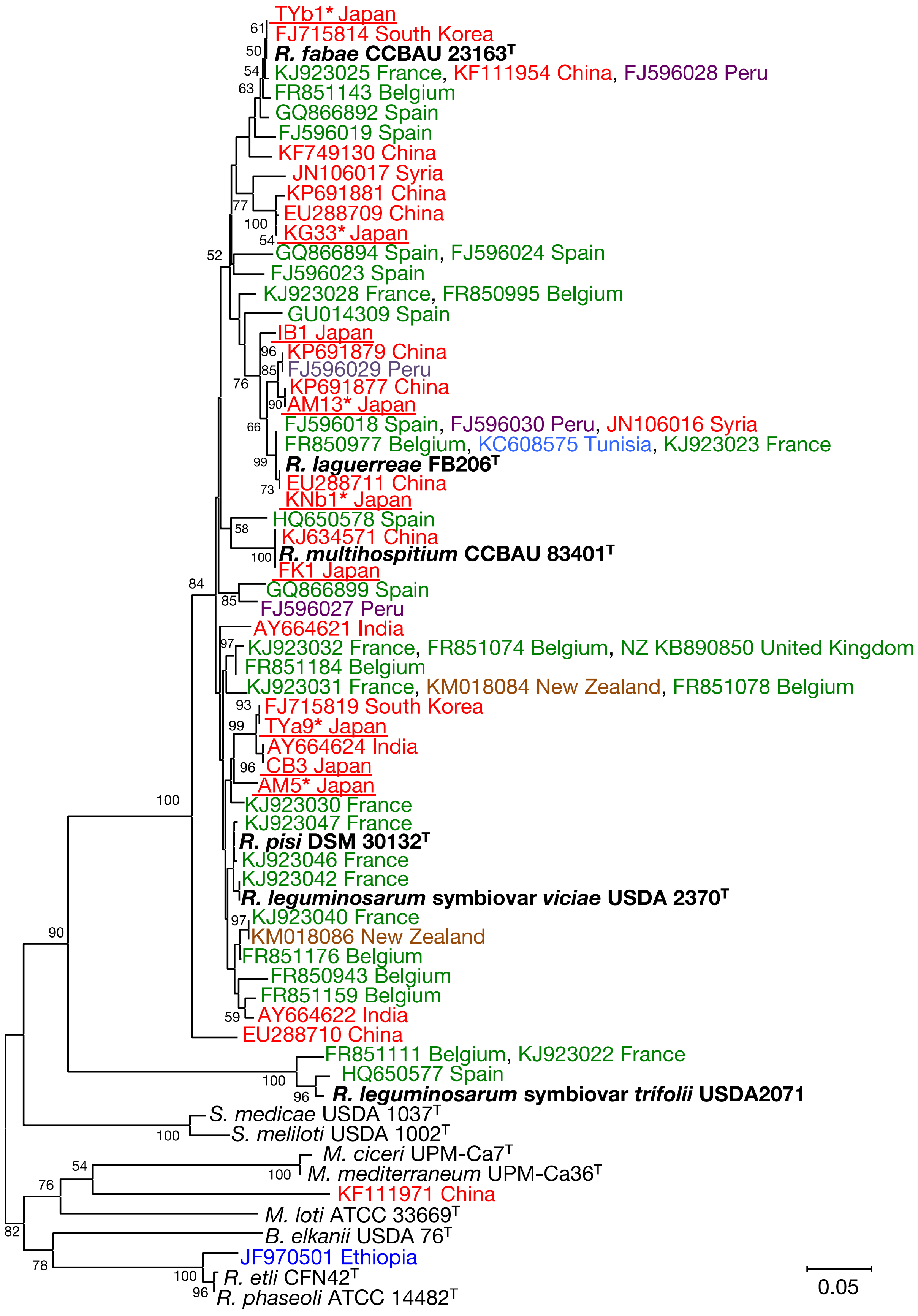

Fig. S3.

(Kun Yuan)
